# ICD-11 Primary Psychotic Disorders: What is New and May be Relevant for Treatment Selection and Outcome?

**DOI:** 10.1192/j.eurpsy.2022.142

**Published:** 2022-09-01

**Authors:** W. Gaebel

**Affiliations:** Heinrich-Heine-Universität Düsseldorf, Psychiatry, Düsseldorf, Germany

**Keywords:** ICD-11, psychotic disorders, treatment

## Abstract

ICD-11 was released by WHO in 2018 and approved by the World Health Assembly (WHA) in 2019. The revision for all chapters was guided by the principles of global applicability, scientific validity and clinical utility. The new chapter for mental health is termed 06 Mental, Behavioural or Neurodevelopmental Disorders (MBND). The ICD-11 with its chapter on Mental, Behavioural or Neurodevelopmental Disorders, its Mortality and Morbidity Statistics (MMS), Coding Tool and Reference Guide, Clinical Descriptions and Diagnostic Guidelines (CDDG), and other tools for translation and implementation offers an innovative approach for individualised diagnosis, treatment and care of people with mental disorders. For supporting the international process of implementation, WHO has installed an International Advisory Group for Training and Implementation of ICD-11 MBND. Development, Concept and Structure of ICD-11 will be presented. Selected changes from ICD-10 to ICD-11 like new diagnostic categories, revision of diagnostic criteria, introduction of dimensional symptom qualifiers or course descriptors, and options for complex coding with regard to their innovative strength, controversial potential and impact on diagnostics, treatment and care will be briefly discussed. National challenges for implementation - partly informed by international field trials, administrative, organisational, educational and training requirements - will be outlined. The new ICD-11 chapter on Schizophrenia or other primary psychotic disorders will serve as an example to discuss potential impact on treatment selection and outcome.

**Disclosure:**

No significant relationships.

